# An one-pot two-step automated synthesis of [^18^F]T807 injection, its biodistribution in mice and monkeys, and a preliminary study in humans

**DOI:** 10.1371/journal.pone.0217384

**Published:** 2019-07-01

**Authors:** Ya-Yao Huang, Ming-Jang Chiu, Ruoh-Fang Yen, Chia-Ling Tsai, Hao-Yu Hsieh, Ching-Hung Chiu, Chi-Han Wu, Ling-Wei Hsin, Kai-Yuan Tzen, Cheng-Yi Cheng, Kuo-Hsing Ma, Chyng-Yann Shiue

**Affiliations:** 1 PET Center, Department of Nuclear Medicine, National Taiwan University Hospital, Taipei, Taiwan; 2 Molecular Imaging Center, National Taiwan University, Taipei, Taiwan; 3 Departments of Neurology, National Taiwan University Hospital, Taipei, Taiwan; 4 Institute of Brain and Mind Sciences, College of Medicine, National Taiwan University, Zhongzheng Dist., Taipei, Taiwan; 5 Department of Psychology, National Taiwan University, Taipei, Taiwan; 6 Graduate Institute of Biomedical Engineering and Bio-informatics, National Taiwan University, Taipei, Taiwan; 7 Department of Radiology, National Taiwan University College of Medicine, Taipei, Taiwan; 8 School of Pharmacy, College of Medicine, National Taiwan University, Zhongzheng Dist., Taipei, Taiwan; 9 PET Center, Department of Nuclear Medicine, Tri-Service General Hospital, Neihu, Taipei, Taiwan; 10 Department of Biology and Anatomy, National Defense Medical Center, Taipei, Taiwan; University of Chicago, UNITED STATES

## Abstract

[^18^F]T807 is a potent tau protein imaging agent. In order to fulfill the demand from preclinical and clinical studies, we developed an automated one-pot two-step synthesis of this potent tau imaging agent and studied its stability, and dosimetry in mice and monkeys. We also conducted a preliminary study of this imaging agent in humans. Using this one-pot two-step method, the radiochemical yield (RCY) of [^18^F]T807 was 20.5 ± 6.1% (n = 15) at the end of bombardment (EOB) in a synthesis time of 70±5 min. The chemical and radiochemical purities were >90% and the specific activities were 151 ± 52 GBq/μmol. The quality of [^18^F]T807 synthesized by this method met the U.S. Pharmacopoeia (USP) criteria. The stability test showed that the [^18^F]T807 injection was stable at room temperature for up to 4 h after the end of synthesis (EOS). The estimated effective dose of the [^18^F]T807 injection extrapolated from monkeys was 19 μSv/MBq (n = 2), while the estimated effective doses of the [^18^F]T807 injection extrapolated from fasted and non-fasted mice were 123 ± 27 (n = 3) and 94 ± 19 (n = 4) μSv/MBq, respectively. This one-pot two-step automated method produced the [^18^F]T807 injection with high reproducibility and high quality. PET imaging and radiation dosimetry evaluation in mice and Formosan rock monkeys suggested that the [^18^F]T807 injection synthesized by this method is suitable for use in human PET imaging studies. Thus, this method could fulfill the demand for the [^18^F]T807 injection in both preclinical and clinical studies of tauopathies, especially for nearby study sites without cyclotrons.

## Introduction

Alzheimer’s disease (AD) is one of the most frequent causes of death and disability worldwide, and has a significant clinical and socio-economic impact. It is the most common form of dementia in individuals over 65 y and accounts for 50–60% of dementia cases. AD is characterized by the degeneration of neurons in the hippocampus and cortex, and the appearance of β-amyloid plaques and neurofibrillary tangles, both of which appear many years before the onset of symptoms of cognitive impairment [1–5]. Although the precise cause of AD remains unclear, it is most likely due to multiple etiologies such as neuronal apoptosis, inflammatory responses, and alterations in various receptors and enzymes. Thus, several PET imaging agents that target multiple mechanisms such as various receptors, enzymes, and β–amyloids have been developed [6, 7] for monitoring the response of AD drug therapy non-invasively and facilitating AD drugs development. Among these, β–amyloid imaging agents have improved the ascertainment of early stages of AD. Unfortunately, the levels of β–amyloid do not correlate as closely to the clinical phenotype as do tau proteins [8–10]. Thus, for the past few years, development of tau imaging agents is among the most active areas of research in molecular imaging of neurodegenerative diseases. This has led to the development of [^18^F]T807 ([^18^F]AV-1451) [11, 12], [^18^F]T808 ([^18^F]AV-680) [13, 14], [^11^C]PBB3 [15], [^18^F]THK5105 [16, 17], [^18^F]THK523 [18–20], [^18^F]THK5117 [16, 21], [^18^F]THK5351 ([^18^F]GE-216) [22], [^18^F]RO6958548, [^11^C]RO6931643, [^11^C]RO6924963 [23–27] and most recently, [^18^F]MK-6240 [28] and [^18^F]PI-2620 [29] as tau imaging agents for clinical studies of AD pathophysiology. Among these, [^18^F]T807 is the most widely used tau imaging agent in clinical studies [12, 30–34]. In addition to several AD clinical studies, [^18^F]T807 has also been applied to studies of chronic traumatic encephalopathy [35], frontotemporal dementia (FTD) [35], Parkinson’s disease [36], and primary progressive aphasia [37].

**Fig 1:**
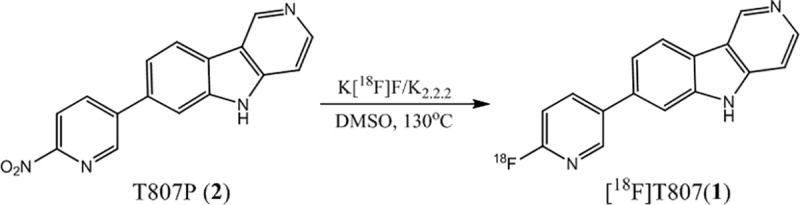
Original one-step radiosynthesis of [^18^F]T807(1).

**Fig 2:**
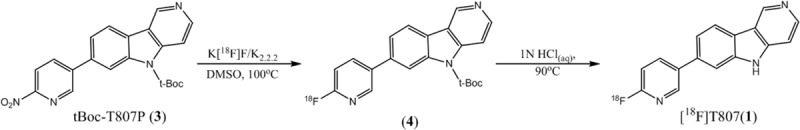
Improved two-step radiosynthesis of [^18^F]T807(1).

[^18^F]T807(**1**) was first synthesized by a one-step method using a modified Siemens Explora radiosynthesis module. Fluorination of its nitro-precursor (T807P, **2**) with [^18^F]Fluoride ([^18^F]F^-^) followed by reduction with Fe powder and purification with HPLC gave [^18^F]T807 in 29–47% yield (EOB) (Fig 1) in a synthesis time of 90–93 min from EOB [11, 12]. However, when [^18^F]T807 was synthesized with a home-built automated synthesis module, its RCY was only 5–10% (EOB) in a synthesis time of 60–70 min from EOB [38]. Meanwhile, Bramoullé *et al*. [39] have modified the semi-preparative HPLC purification conditions without using Fe powder as a reducing agent and successfully produced [^18^F]T807 with TRACERlab FX_FN_ Pro synthesizer in 14% yield (EOB) in a synthesis time of 75 min from EOB. However, there was no mention of either the chemical or radiochemical purity of the [^18^F]T807 product synthesized by this method. Recently, Shoup *et al*. [40] have reported an improved one-step synthesis of [^18^F]T807 using a *t*-Boc-protected nitro-precursor (*t-*Boc-T807P or AV-1628, **3**) and *on-line* acidic de-protection of *t*-Boc-protecting group, resulting in a RCY of 16–25% (EOB) in a synthesis time of 60 min from EOB (Fig 2) (Method A). However, we have found that this *on-line* acidic de-protection of the *t*-Boc-protecting group was somewhat difficult to reproduce.

In order to facilitate its widespread use for tauopathies and traumatic brain injuries [41, 42], it is imperative to have a reliable, fully automated high yield and GMP-compliant manufacturing process method available for the production of the [^18^F]T807 injection **(1)**. Thus, we have adopted Shoup’s method [40] with minor modifications to fully automate the synthesis of the [^18^F]T807 injection under GMP-compliance using a TRACERlab FX_FN_ module (GE Healthcare, Milwaukee, WI) in high quality and high reproducibility, and study its whole-body biodistribution and dosimetry estimations in ICR mice, Formosan rock monkeys and in humans. We herein report a GMP-compliant high reliability fully automated synthesis of [^18^F]T807 using a TRACERlab FX_FN_ module along with its whole-body biodistribution in ICR mice and Formosan rock monkeys, and the validation of this radiotracer in human. Part of this work has been present in abstract form [43].

## Material and methods

### Chemicals and reagents

**Fig 3:**
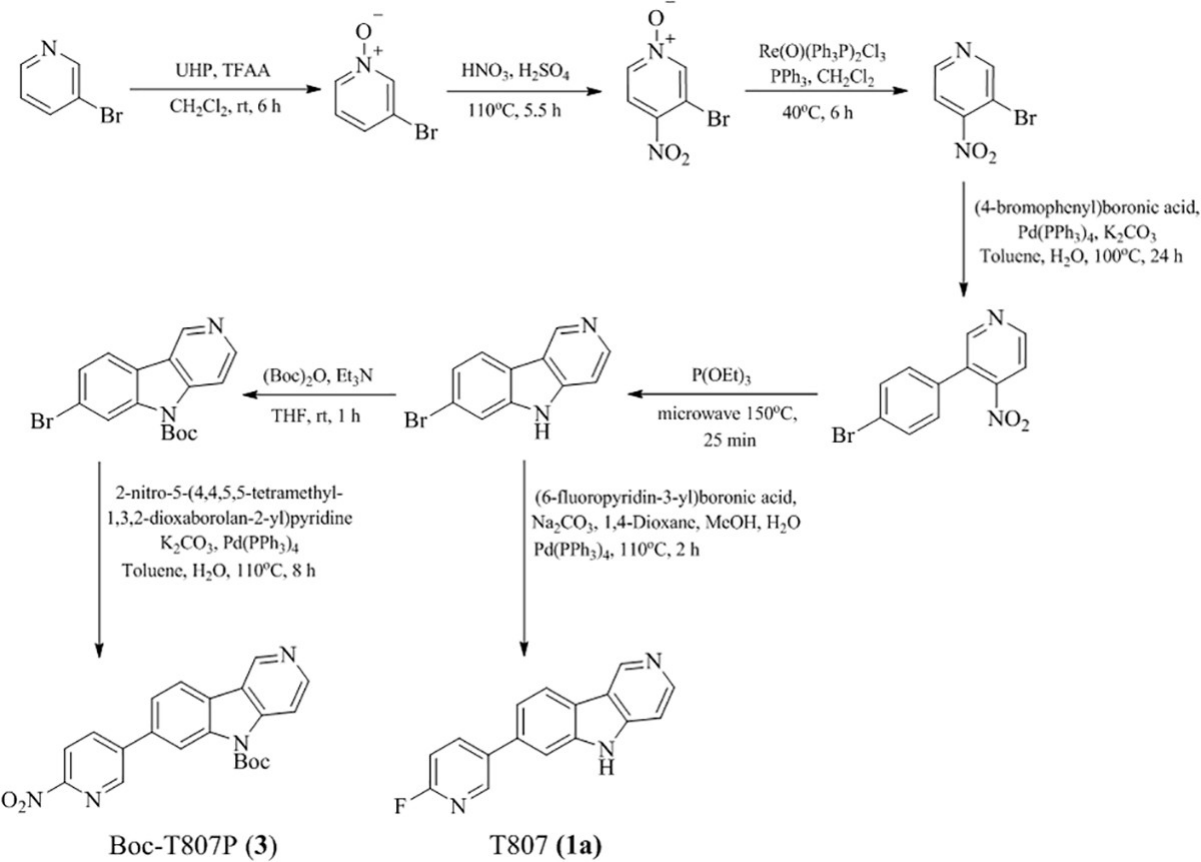
Synthesis of precursor (3) and the non-radioactive standard (1a) of [^18^F]T807 (1).

All chemicals and reagents were obtained from commercial vendors and were used as received without further purification. All Sep‐Pak cartridges were obtained from Waters (Milford, MA, USA) and highly enriched (> 98.0 Atom%) [^18^O]H_2_O was purchased from Huayi Isotopes Co. (Changshu, China). Aqueous [^18^F]F^−^ was produced in our PET Trace cyclotron (GE Medical Systems, Uppsala, Sweden) via an ^18^O(p, n)^18^F nuclear reaction. The precursor (*tert*-butyl-7-(6-nitropyridin-3-yl)-5*H*-pyrido[4,3-*b*]indole-5-carboxylate, **3**) and the non-radioactive authentic standard T807 (**1a**) were synthesized by literature method [44] with minor modifications as shown in Fig 3. The identity of **3** was confirmed by ^1^H-NMR (S1 Fig), ^13^C-NMR (S2 Fig), and mass spectra (S3 Fig). The chemical purity of compounds **3** was determined by reverse-phase high-performance liquid chromatography, and was higher than 95% (S4 Fig) (please see Supporting Information).

### Fully automated production of the [^18^F]T807 injection using a FX_FN_ module

**Fig 4:**
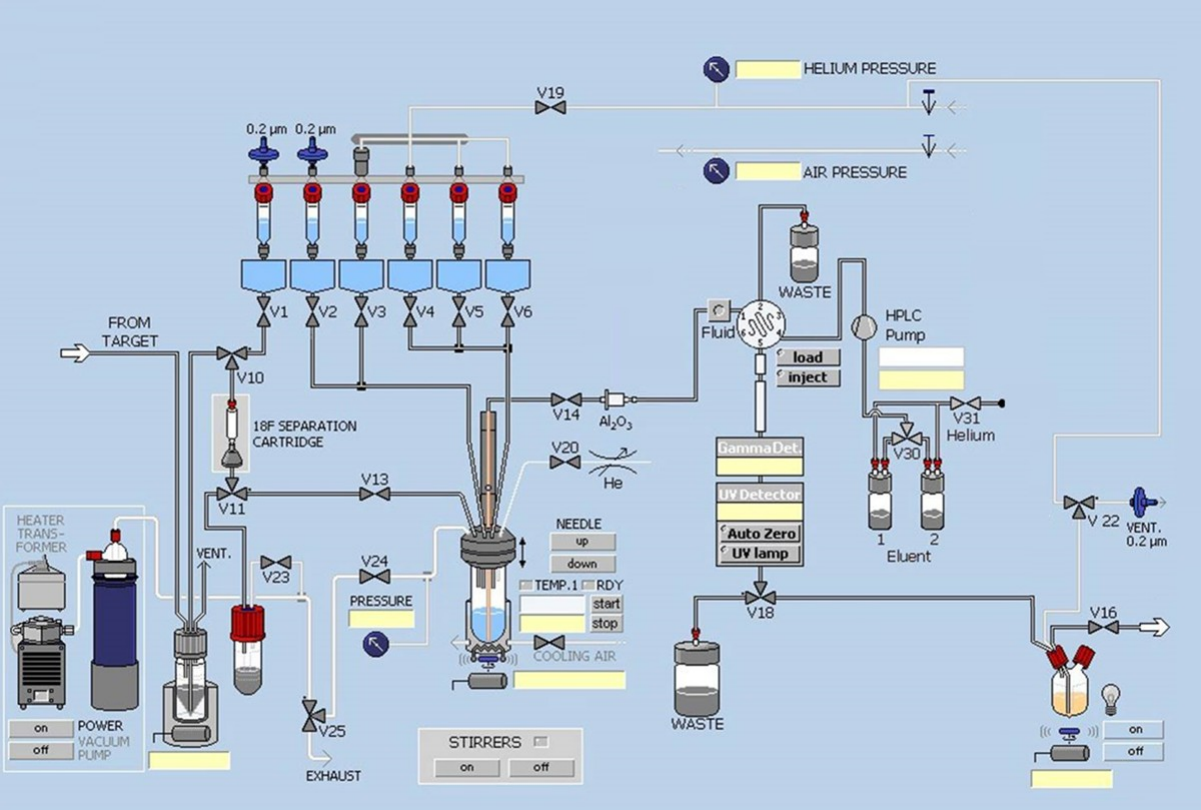
Modified TRACERLab FX_FN_ module for [^18^F]T807 radiosynthesis.

Prior to delivery of [^18^F]fluoride to the TRACERLab FX_FN_ synthesis module, each vial was filled with appropriate solvent and reagent as shown in Fig 4.

Vial 1: K_2_CO_3_ (4.7 mg in 0.25 mL H_2_O) and K_2.2.2._ (14.7 mg in 0.25 mL ACN).
Vial 2: Acetonitrile (ACN) (1.0 mL).
Vial 3: Precursor (1mg) in 0.5 mL DMSO.
Vial 4: 0.4 mL of 1 N HCl_(aq)_.
Vial 5: 0.5 mL of 2 M NaOAc_(aq)_.
Vial 6: 0.35 mL of EtOH/21 mM sodium phosphate (8/92).
Product bottle: 0.13 mL of 7% NaHCO_3(aq)_.

V14 was directly connected to a Sep-Pak Alumina N Plus Long cartridge and then connected to the tubing passing through the fluid detector for semi-preparative HPLC purification.

The automated synthesis module was operated in the following sequence:

1. At the end of bombardment, aqueous [^18^F]F^−^ in 1.4 mL of [^18^O]H_2_O was transferred from the target into a [^18^O]H_2_O collection vial with helium purge.
2. Opened V10; aqueous [^18^F]F^−^ was passed through a Sep-Pak QMA Plus Light cartridge and V11 under vacuum. [^18^O]H_2_O was recovered in the [^18^O]H_2_O recovery vial and the [^18^F]F^−^ was trapped on a Sep-Pak QMA Plus Light cartridge.
3. Opened V1, V13, and V24. K_2_CO_3_/K_2.2.2_ solution in Vial 1 was passed through V1, V10, QMA, V11, and V13. [^18^F]F^−^ was collected in Reaction Vessel.
4. Closed V1, V13, and V14. Opened V24 and evaporated the solution in Reaction Vessel at 110°C for 3 min under a stream of helium from V20.
5. Flushed ACN in Vial 2 into Reaction Vessel with helium. Closed V2, V13, and V14. Opened V24, and performed azeotropic distillation of the remaining H_2_O with a stream of helium from V20.
6. Flushed precursor solution in Vial 3 into Reaction Vessel with helium. Closed V2, V3, V13, V14, V20, and V24. Heated the solution at 130°C for 10 min.
7. Flushed HCl_(aq)_ in Vial 4 into Reaction Vessel with helium. Closed V4, V13, V14, V20, and V24. Heated the solution at 90°C for 10 min.
8. Flushed NaOAc_(aq)_ in Vial 5 and EtOH/sodium phosphate in Vial 6 into Reaction Vessel with helium. Closed V5, V6, V13, V14, V20, and V24. Stirred the solution for 1 min at 50°C.
9. Opened V14, passed the reaction mixture in the Reaction Vessel through a Sep-Pak Alumina N cartridge with a stream of helium from V20 and immediately injected it into semi-preparative HPLC (Phenomenex Luna(2) HILIC, 5 μm, 10 × 250 mm, 0.1% HCl_(aq)_, 254 nm, 3 mL/min).
10. At approximately 18 min post-injection, the [^18^F]T807 peak was collected for 3 min and the solution was transferred through V18 into the product bottle, which contained 7% NaHCO_3(aq)_.
11. Opened V16 and flushed the [^18^F]T807 solution in the final product bottle, which contained 0.13 mL of 7% NaHCO_3(aq)_ and 9 mL of 0.1% HCl_(aq)_, through a 0.2 μm sterile filter (Millex LG; Millipore, Billerica, MA, USA), into a sterile multi-injection vial with a stream of helium from V22.

### Quality control and stability tests of the [^18^F]T807 injection

The quality control (QC) tests of the [^18^F]T807 injection were performed in accordance with the USP for radiopharmaceuticals, which include visual inspection, pH, half-life of radionuclide, radionuclidic purity, radiochemical purity, chemical purity, residual K_2.2.2_, residual solvents, bacterial endotoxins, filter integrity, and sterility test as tabulated in Table 1.

**Table 1:** Production and QC results of three consecutive productions of the [^18^F]T807 injection (n = 3).

**Batch Production**		**Production Results**		
		**Run 1**	**Run 2**	**Run 3**
**Starting Activity (GBq)**		77.4	32.3	92.9
**Product Activity GBq)**		5.3	1.7	6.8
**Final Volume (ml)**		9	9	9
**Synthesis Time (min)**		57	61	66
**Specific Activity (GBq/μmol) (EOB)**		542	323	408
**QC Test**	**Acceptance Criteria**	**QC Results**		
**Visual inspection**	**Clear, colorless solution**	Pass	Pass	Pass
**pH value**	**5–8**	7	7	7
**Residual K**_**2.2.2**_ **(**μ**g/mL)**	**<50**	<50	<50	<50
**Radionuclidic Purity (%)**	**>99.5**	99.99	99.99	99.98
**Half-life (min)**	**110±5**	115	114	113
**Radiochemical Identity (min)**	**∣R**_**t**_**-R**_**t(Reference)**_**∣≤0.5**	0.25	0.10	0.23
**Radiochemical purity (%)**	**>90**	91.47	92.38	93.12
**Residual solvent analysis**	**Acetone<0.5%**	0.0116	0.0069	ND
	**ACN<0.04%**	ND	0.047	ND
	**DMSO<0.5%**	0.0114	ND	0.0112
	**EtOH<0.5%**	0.0069	0.0113	0.2469
**Residual [**^**18**^**F]F**^**-**^ **(%)**	**<5**	0.12	0.26	0.26
**Bacterial endotoxins (EU/ml)**	**<17.5**	<5	<5	<5
**Filter integrity test (psi)**	**>50**	59	58	56
**Sterility test**	**Sterile**	Sterile	Sterile	Sterile

The radiochemical and chemical purities of the [^18^F]T807 injection were analyzed with a HPLC system (Agilent 1100 series) equipped with a Bioscan FC3300 flow count radioactivity detector (2” × 2” pinhole) and an UV detector (254 nm) using a Phenomenex Luna HILIC column (5 μm, 4.6 × 250 mm) with 10% EtOH (pH = 2, adjusted with HCl) as mobile phase and a flow rate of 1 mL/min. The radiochemical purity of [^18^F]T807 was further analyzed with radio TLC (Silica gel 60 F254 plate, NH_4_OH/MeOH/CH_2_Cl_2_ (0.8/5/95)) and detected with a Raytest miniGita radio-TLC scanner (Raytest, Straubenhardt, Germany).

The residual organic solvents in the [^18^F]T807 injection were analyzed by gas chromatography (GC) equipped with a flame ionization detector (FID) and an Agilent Column (HP Fast Residual Solvent chromatography column, 30 m × 0.53 mm × 1 μm). Nitrogen was used as a carrier gas with a flow rate of 2 mL/min. The split/splitless injector was set at 20:1, the initial oven temperature was kept at 40°C for 1 min followed by an increase of temperature at 14°C/min to 110°C and then at 30°C/min to 125°C. The oven temperature was kept at 125°C for 2.5 min followed by an increase of temperature at 47.5°C/min to 220°C and kept at that temperature for 4 min. Hydrogen and air, at 35 and 250 mL/min, respectively, were used in the GC/FID, with nitrogen (25 mL/min) as a make-up gas. The software Gina Star (Raytest, Straubenhardt, Germany) was used for data acquisition.

Measurement of bacterial endotoxins was carried out using a Limulus amoebocyte lysate (LAL) test with Kinetic-QCL method (Lonza, Walkersville, MD, USA) and sterility test was performed with the proven method meeting the requirement of USP.

The stability of the [^18^F]T807 injection at room temperature (18°C~25°C) was monitored with both TLC and HPLC as described above for up to 4 h after EOS.

### Whole-body biodistribution of the [^18^F]T807 injection in mice and monkeys

Male ICR mice (20–30 g, 6–8 weeks old) and male Formosan rock monkeys (*Macaca cyclopis*) (5 and 7.9 kg, n = 2) were used for this study. The animal study protocols of mice and monkeys used for this study were approved by the Laboratory Animal Center of National Taiwan University (NTU) and National Defense Medical College (NDMC), respectively. Both animal centers have been granted full accreditation from the Association for Assessment and Accreditation of Laboratory Animal Care International (AAALAC) since 2007. The animals were housed and handled according to institutional guidelines.

ICR mice were obtained from NTU College of Medicine Laboratory Animal Center and housed in the animal facilities at Department of Nuclear Medicine of National Taiwan University Hospital (Taipei, Taiwan) at a constant temperature of 26 ± 2°C, humidity of 30~70%, and a controlled light/dark cycle (light from 7:00 AM to 7:00 PM). Mice were maintained on a complete pellet diet and tap water for a period of 1 week prior to the studies. To compare the effect of fasting on [^18^F]T807 whole body bio-distribution, the mice were either fasted for at least 8 hours (n = 3) or non-fasted (n = 4) as the control counterparts.

The monkeys were bred at a temperature of 21~25 ^o^C, humidity of 50~75%, ventilation of 8~15/hour, illumination from 7:00 AM to 7:00 PM, noise < 65 dB, and sanitation, to escalate the homeostasis of animals in NDMC Animal Center (Taipei, Taiwan).

During scanning, mice were anesthetized with 2% isoflurane in oxygen and the monkeys were immobilized with ketamine (2 mg/kg), anesthetized with 2% isoflurane via an endotracheal tube and administered with atropine sulfate (2 mg IM) to minimize secretions during the course of the experiment. Body temperature was kept constant at 37°C with an additional blanket. The heart rate, *p*O_2_ and *p*CO_2_ were checked every 10 min and kept in the normal range throughout the imaging sessions. Whole-body transmission and emission scans of ICR mice and Formosan rock monkeys were acquired with a small-animal Argus PET/CT scanner (SEDECAL, Madrid, Spain) and a BIOGRAPH PET/CT scanner (Biograph Duo, Siemens, USA), respectively. For mice studies, after the [^18^F]T807 injection (9 ± 3 MBq), dynamic sinograms were produced for 90 min with 2 x 10 s, 2 x 20 s, 4 x 60 s, 2 x 240 s, 2 x 600 s, and 1 x 720 s frames. For the monkeys’ studies, after the [^18^F]T807 injections (107 MBq and 89 MBq), a low dose CT scan (130 kVp, 50 mAs), a series of eight whole-body PET scans were performed (15, 55, 95, 110, 155, 170, 215 and 230 min) post-injection. Total acquisition time was 240 min. Each scan covered the monkey’s body from the head to the thigh and consisted of five to six bed positions depending on the size of the monkey. In each bed position, data was acquired for 2 min in 3D mode. The data was then reconstructed by OSEM (Ordered-Subsets Expectation-Maximization) on a 128 × 128 matrix (slice thickness, 5 mm), 2 iterations eight subsets, 3 mm FWHM Gaussian filter, and corrected by photon attenuation using the CT scan.

The image data analysis and residence time calculation were performed by the method reported previously [45–47]. Each CT and PET whole-body image was successively loaded to the PMOD 3.0 software (PMOD technologies, Switzerland; www.pmod.com) to generate the fusion images. The regions of interest (ROIs) of these organs were manually drawn as precisely as possible on the organ itself, on each horizontal slice. Organs with high uptake of [^18^F]T807, such as brain, lungs, and heart were easily identified and outlined. Organs with low uptake of [^18^F]T807, or those with complicated anatomy such as liver, gallbladder, kidneys, and bladder, were processed manually. ROIs were adjusted (slice by slice) for organ movement (such as intestine peristalsis) between time frames. In order to evaluate the significant tracer accumulation in large intestine of mice, the ROI of the large intestine was defined as the sum of [^18^F]T807 uptakes for all time points. The activity in each organ was non-decay-corrected and expressed as standard uptake value (SUV) (n = 2, Mean for monkeys; n = 4, Mean± S.D. for mice).

### Radiation dosimetry evaluation of the [^18^F]T807 injection from animal images

To calculate the absorbed radiation dose of each organ as well as the effective dose, the injected dose of the [^18^F]T807 injection and the non-decay-corrected TACs of all source organs of two monkeys and four mice were individually entered into an Excel spreadsheet, and the data was processed individually for each animal. The difference between the injected dose and the sum of the whole-body radioactivity (assuming that there was no excretion) plus the radioactivities of the above-mentioned organs was taken as the TAC of the remainder.

The residence time (h) of the selected organ was calculated as the area under the TACs of the source organ from time zero to infinity over the initial total body activity (5 half-lives of ^18^F, i.e. approximately 550 min). The area under the TAC curve of each organ was generated using the following strategy, including trapezoidal integration of the first four TAC data points through the origin and exponential decline of the four remaining TAC data points to infinity such that the residence times of these organs were obtained [45].

The human dosimetry was estimated from both mice and monkeys biodistribution data. The organ weight and body mass were used for allometric scaling [47]. That is, the residence time in each organ was converted to the corresponding human value by multiplication with a factor to scale organ and body weights (in kg) as (w_m,b_/ w_m,o_)(w_h,o_/ w_h,b_), where w_m,b_ was the monkey body weight, w_m,o_ was the monkey organ weight, w_h,b_ was the human body weight, and w_h,o_ was the human organ weight. However, the w_m,o_ values of Formosan rock monkeys *(Macaca cyclopis*) were unavailable. We have instead used the w_m,o_ values of rhesus monkey (*Macaca mulatta*) in our calculations. Both monkeys’ organ weights [48, 49] and humans’ organ weights [50] were obtained from the literature. The residence time of [^18^F]T807 in the rest of the body was obtained by subtracting the total organ residence time from the reciprocal of the ^18^F decay constant.

Absorbed radiation doses were calculated from the residence times in all source organs for each monkey by entering the information into the Java-based OLINDA 1.0/EXM computer program [51] using the model for a 70-kg adult male and female phantom.

### PET/CT imaging of the [^18^F]T807 injection in an AD patient

#### Subject

The representative subject (male, age = 61) in this study was noted to have visuospatial problem and impaired memory function. In addition, he had completed more than 16 years of education and worked as a medical professional. His Mini-Mental State Exam (MMSE) [52] was 24, which was mildly impaired for his education and occupation. The neuropsychological test showed impaired verbal (word sequence learning) and non-verbal memory (Benton Visual Retention Test), impaired language function (object naming and semantic verbal fluency), visual perceptual function (3D block deign), and executive function (Wisconsin Card Sorting Test). Based on the NIA-AA workgroups in 2011 [53], the clinical diagnosis was early onset AD, probably a posterior cortical atrophy variant.

#### PET imaging

Imaging with [^18^F]T807 was performed with GE Healthcare Discovery ST4 PET/CT scanner (2D mode, 47 image planes, 15.0 cm axial field of view). After 80–100 min of intravenous injection of approximately 185 MBq (specific activity = 151 GBq/μmol) of [^18^F]T807, PET images of human brain were acquired over 20 min and processed by the method reported previously [12, 54].

## Results and discussion

### GMP production of the [^18^F]T807 injection

**Fig 5:**
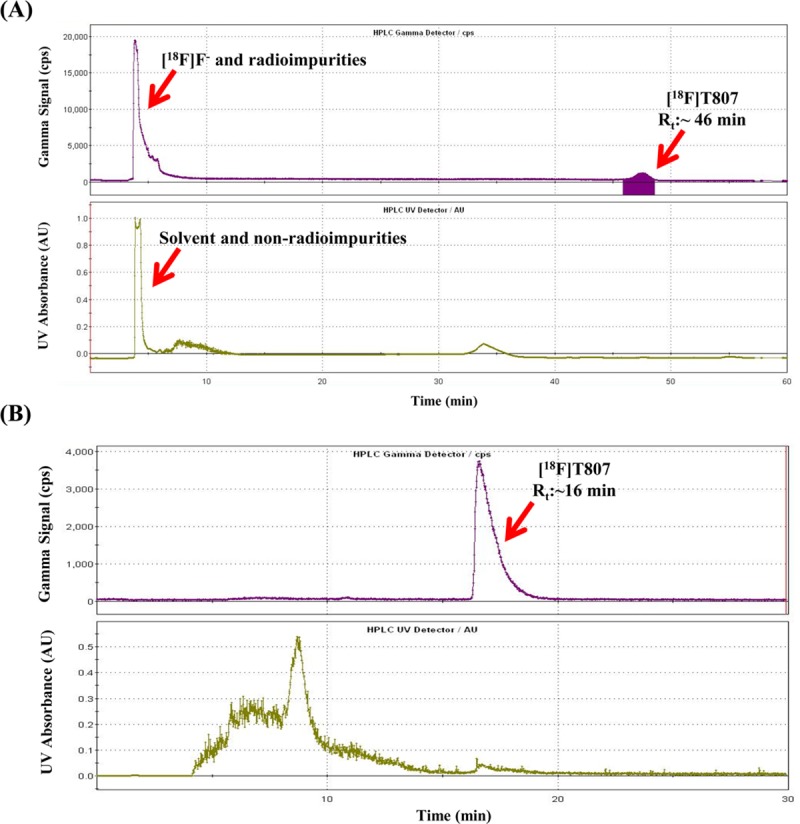
Representative semi-preparative HPLC chromatograms of [^18^F]T807 synthesized by Method A (on-line one-step *t*-Boc deprotection and HPLC purification, Waters HSS T3, 5 μm, 10 × 250 mm, 18% EtOH(pH 2.0, adjusted by HCl), 5.0 ml/min, 254 nm) (A) and Method B (two-step *t*-Boc deprotection and HPLC purification, Phenomenex Luna(2) HILIC, 5 μm, 10 × 250 mm, 0.1% HCl_(aq)_, 254 nm, 3 mL/min) (B).

[^18^F]T807 **(1)** is a potent tau imaging agent and has been synthesized by several methods in various radiochemical yields and radiochemical purities [11, 12, 38–40]. Initially, [^18^F]T807 was synthesized by nucleophilic fluorination of the nitro-precursor (T807P, **2**) with [^18^F]F^−^, followed by reduction with Fe/HCl and purification with HPLC, to give the desired product in 29–47% yield (EOB) (Fig 1) [12]. However, this synthesis sequence was somewhat complicated. Thus, several improved methods have been reported [38–40]. Among these, Shoup *et al*. have developed a new method to synthesize [^18^F]T807 by radiofluorination of its t-Boc protected nitro- precursor (tBoc-T807P, **3**) with [^18^F]F^-^ followed by *on-line* de-protection with HCl/HPLC to give [^18^F]T807 in 16–25% (EOB) in a synthesis time of 60 min (Fig 2) [40]. We have tried to adapt this method to synthesize [^18^F]T807 for our pre-clinical and clinical studies. However, we encountered some difficulty in repeating this method. For example, when the reaction mixture was injected into HPLC, we have found that the retention time (Rt) of [^18^F]T807 chromatogram (Rt = 40~49 min, n = 3, as shown in Fig 5(A)) was significantly different from that of the reported value (22~24 min). In addition, a significant amount of radioactivity was lost during *on-line* de-protection with HCl/HPLC purification, SPE post-formulation and sterile filtering, resulting in low overall RCYs of [^18^F]T807 (1~3%, EOS, n = 3) compared to Shoup’s data (14 ± 3%, EOS). Although using the *on-line* de-protection of intermediate ([^18^F]**4**) and purification of final product could simplify the production process for the [^18^F]T807 injection, the reproducibility of this method is something to be concerned about. It has been reported that this de-protection route may rely on several factors such as the strength and stoichiometry of the acid used, and the flow rate of HPLC eluent, etc. [55]. As a result, we have chosen to use HCl for *in-situ* de-protection of the intermediate followed by purification of [^18^F]T807 with HPLC (Phenomenex Luna (2) HILIC column; 5 μm, 10 × 250 mm; eluent: 0.1% HCl_(aq)_; flow rate: 3 ml/min). The semi-preparative HPLC chromatogram of [^18^F]T807 purified by this method was depicted in Fig 5(B). Using this modified purification method, we are able to produce [^18^F]T807 routinely with a FX_FN_ Module in 20.5 ± 6.1% yield (EOB, n = 15) in a synthesis time of 70 ± 5 min from EOB. Typically, start with 27.4 ± 16.6 GBq of [^18^F]F^−^, 3.4 ± 2.3 GBq of the [^18^F]T807 injection was produced at EOS. Both the chemical and radiochemical purity of the [^18^F]T807 injection were >90% with a specific activity of 151±52 GBq/μmol (n = 15, EOS), and has been used for pre-clinical and clinical studies.

After the completion of this study, a similar synthetic method with a home-made module was used to synthesize [^18^F]T807 in 20–30% yield (EOB) in a synthesis time of 60–70 min from EOB [38]. However, this synthetic method required solid phase extraction and reformulation of [^18^F]T807 after HPLC purification. Most recently, a new *t*-Boc-protected precursor (AV-1622) with trimethylammonium as a leaving group has been used to synthesize [^18^F]T807 in 25~55% yield (EOB) in a synthesis time of 45~60 min [56–60]. The applicability of this new precursor for routine automated production of [^18^F]T807 has not yet been proven.

### Quality control and stability test of the [^18^F]T807 injection

**Fig 6:**
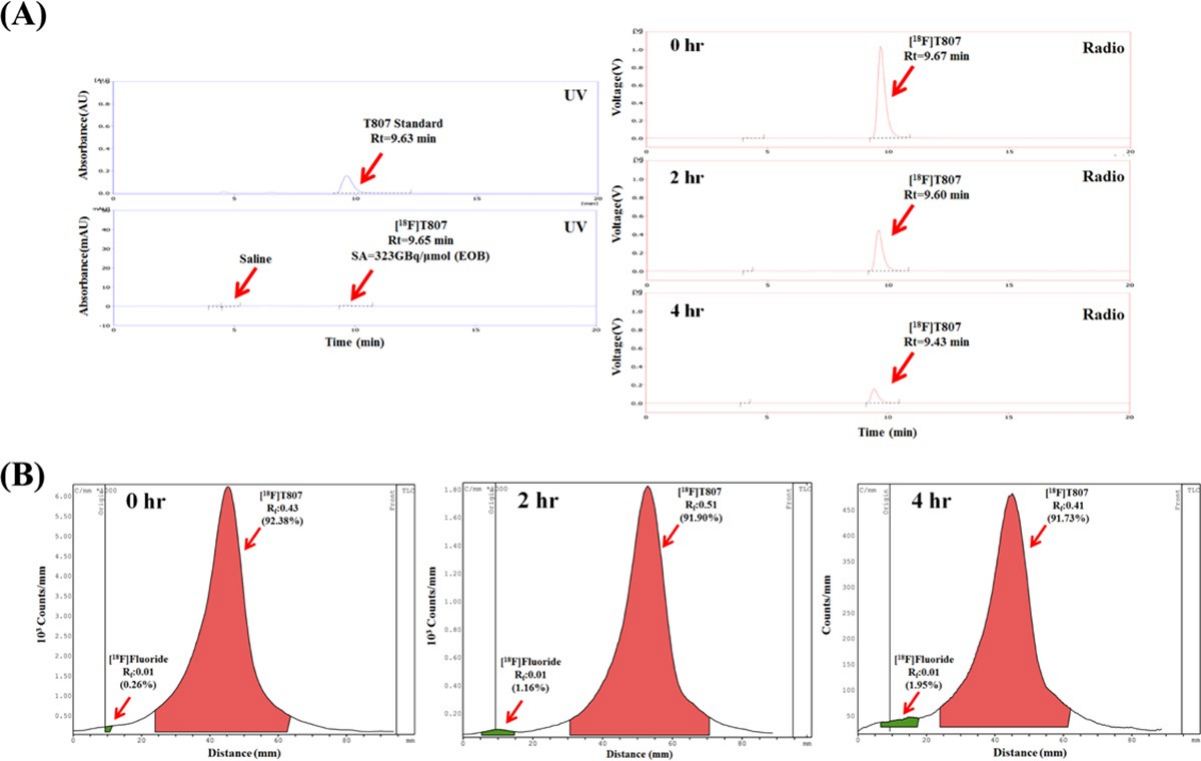
Representative analytical radio-HPLC (A) and radio-TLC chromatograms (B) of [^18^F]T807 at 0, 2 and 4 hrs after EOS.

The QC test results of three batches of validation runs of the [^18^F]T807 injection are tabulated in Table 1. The quality of the [^18^F]T807 injection synthesized by this method met the USP criteria: The appearance of the [^18^F]T807 injection was clear, the HPLC retention time of the [^18^F]T807 injection was approximately10 min (Fig 6(A)), the retardation factor (R_f_) values of [^18^F]F^−^ and the [^18^F]T807 injection were approximately 0.0 and 0.4, respectively (Fig 6(B)), the radiochemical purity of the [^18^F]T807 injection was greater than 90% (Fig 6), and the concentrations of residual solvents in the [^18^F]T807 injection were Acetone < 0.5%, DMSO < 0.5%, EtOH < 0.5%, and ACN < 0.04% (Table 1). The stability test showed that the [^18^F]T807 injection was stable at room temperature for up to 4 h after EOS (Table 2).

**Table 2:** Stability of three consecutive productions of the [^18^F]T807 injection (n = 3).

Test	Acceptance Criteria	Elapsed time (hr)	Run 1	Run 2	Run 3
**Radiochemical purity (%)**	**> 90%**	0	91.5	92.38	93.12
		2	90.5	91.90	91.86
		4	90.6	91.73	91.08
**Residual [**^**18**^**F]fluoride (%)**	**< 5%**	0	0.1	0.26	0.26
		2	1.4	1.16	0.58
		4	2.5	1.95	0.66

### Whole-body biodistribution of the [^18^F]T807 injection in mice and monkeys

The [^18^F]T807 injection has been demonstrated as a potent tau imaging agent in humans [12, 61–63]. However, its radiation dosimetry in humans was not available until very recently [64]. In order to fulfill the Taiwanese Food and Drug Administration (TFDA) requirements, we studied its organ radiation dose burden in animal models and extrapolated it to humans before human PET studies are undertaken.

*Ex vivo* studies of [^18^F]T807 in mice showed that [^18^F]T807 cleared rapidly from the brain and had high uptake in the kidneys, the bladder, and the liver, and relatively low uptake in muscle and bone [11].

**Fig 7:**
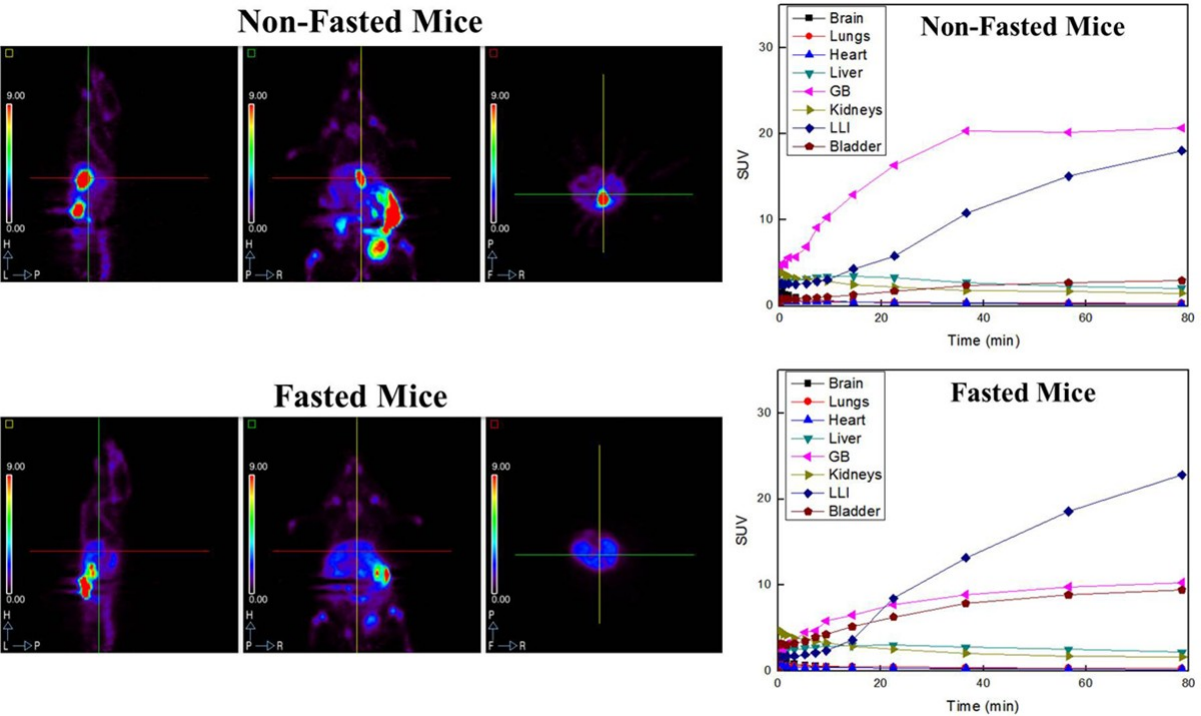
Representative whole-body biodistribution of [^18^F]T807 in normal mice at last time-frame post injection and non-decay-corrected time-activity curves of [^18^F]T807 in various organs of non-fasted mice (n = 4) and fasted mice (n = 3).

**Fig 8:**
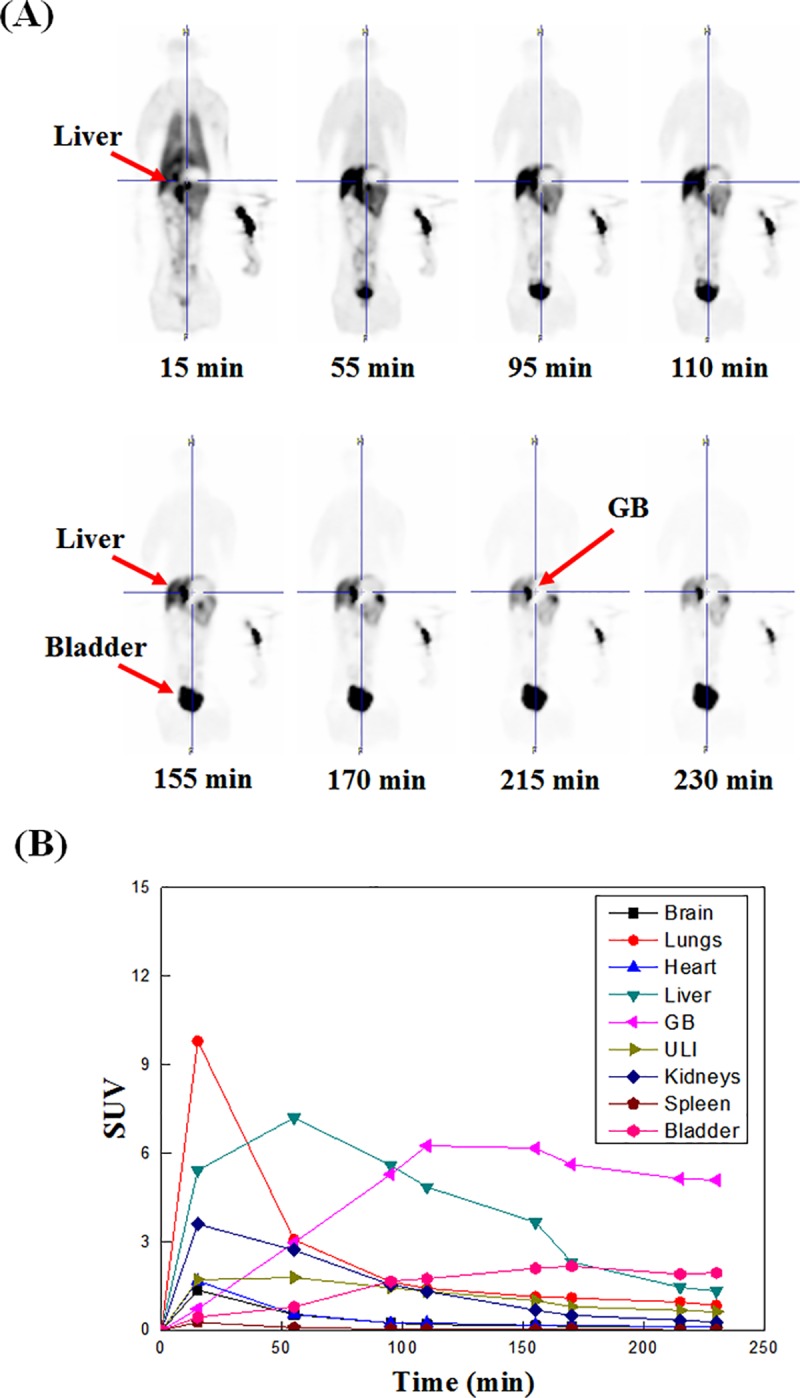
Typical sagittal view of [^18^F]T807 whole-body images of a male Formosan Rock monkey at different time points (A) and Non-decay-corrected time-activity curves of [^18^F]T807 in various organs of monkeys (n = 2) (B).

PET studies of the [^18^F]T807 injection in mice and monkeys showed that [^18^F]T807 did not produce overt adverse effects, and its uptake in gallbladders of fasted and non-fasted mice varied significantly (Fig 7). It also showed that the uptake of radioactivity in mice GB and LLI were high and increased with time (Fig 7), while its uptake in the monkey’s lung, liver, and kidneys were high in the beginning and gradually declined thereafter (Fig 8). The radioactivity in the gallbladder and bladder increased with time, suggesting that [^18^F]T807 may be eliminated via hepatobilliary and renal systems, which was in agreement with Choi’s results [64].

### Radiation dosimetry and toxicity of the [^18^F]T807 injection

The organ radiation absorbed dose studies of the [^18^F]T807 injection were carried out in both fasted and non-fasted ICR mice and Formosan rock monkeys. The residence times of selected organs in mice from the TACs of both the *ex vivo* biodistribution studies [11] and the PET imaging are tabulated in Table 3.

**Table 3:** Residence times (hr) of selected organs from the biodistribution data and microPET imaging of mice and monkeys.

Source Organ	Residence times (hr)			
	*Ex Vivo* Data	*In Vivo* Imaging		
	Mice	Fasted Mice (n = 3)	Non-fasted Mice (n = 4)	Fasted Monkey (n = 2)
**Brain**	0.011	0.011 ± 0.003	0.024 ± 0.008	0.023 ± 0.008
**Lung**	-	0.044 ± 0.009	0.056 ± 0.030	0.111 ± 0.027
**Heart**	-	0.003 ± 0.001	0.003 ± 0.001	0.035 ± 0.002
**Liver**	0.046	0.025 ± 0.004	0.033 ± 0.017	0.199 ± 0.134
**Muscle**	0.013	-	-	-
**Bone**	0.005	-	-	-
**Spleen**	-	-	-	0.005 ± 0.002
**GB**	-	0.251 ± 0.212	0.525 ± 0.387	0.477 ± 0.031
**LLI**	-	1.373 ± 0.354	1.075 ± 0.322	0.178 ± 0.030
**Kidneys**	0.019	0.036 ± 0.034	0.029 ± 0.026	0.045 ± 0.004
**Bladder**	-	0.352 ± 0.167	0.148 ± 0.087	0.110 ± 0.008
**Remainder**	2.547	0.546 ± 0.569	0.747 ± 0.659	1.457 ± 0.150

Using these residence times, the individual organ doses in humans as extrapolated from the mice are tabulated in Table 4 [64]. The results from microPET imaging showed that the target organs of the [^18^F]T807 injection were the gallbladder, LLI, and bladder, and the estimated effective doses extrapolated from fasted and non-fasted mice were 123.0 ± 27.4 μSv/MBq (n = 3) and 93.7 ± 19.0 μSv/MBq (n = 4), respectively. In contrast, the results from *ex vivo* studies, which include only kidneys, liver, brain, muscle, and bone [11], showed that the estimated effective dose was approximately 14 μSv/MBq (Table 4). The exact reason(s) for the discrepancy between *ex vivo* and *in vivo* studies is unclear, but omission of some mice organs in *ex vivo* studies may account for this difference. Although partial volume effects in microPET imaging caused by the very small mice organ size may contribute to the underestimation of the estimated effective dose, microPET imaging is still regarded as a useful tool for absorbed dose estimations in humans.

**Table 4:** Radiation dosimetry estimates of the [^18^F]T807 injection extrapolated from the mice and monkey biodistribution data.

Target Organ	Radiation Dosimetry (μGy/MBq)				
	*Ex Vivo* Data	*In Vivo* Imaging			
	Mice	Fasted Mice (n = 3)	Non-Fasted Mice (n = 4)	Fasted Monkey (n = 2)	Human [64]
**Adrenals**	14.7	7.1 ± 2.0	9.7 ± 0.5	16.3 ± 0.7	18.7
**Brain**	4.6	2.4 ± 1.2	5.3 ± 1.5	5.5 ± 1.5	12.7
**Breasts**	12.2	3.0 ± 2.0	4.7 ± 2.0	7.7 ± 0.2	9.8
**Gallbladder**	15.7	399.4 ± 330.5	632.4 ± 635.1	763.5 ± 46.0	44.9
**LLI wall**	16.0	814.7 ± 204.5	626.3 ± 153.1	11.7 ± 0.2	13.0
**Small Intestine**	17.6	31.4 ± 4.7	28.5 ± 4.7	19.2 ± 2.2	31.6
**Stomach**	15.9	10.0 ± 0.9	11.8 ± 0.0	14.0 ± 0.2	13.6
**ULI wall**	17.0	20.4 ± 3.1	21.9 ± 5.7	66.3 ± 40.0	16.8
**Heart wall**	16.2	5.0 ± 2.2	7.2 ± 1.8	18.9 ± 0.4	24.6
**Kidneys**	18.6	29.2 ± 19.3	25.9 ± 11.1	71.4 ± 48.9	34.9
**Liver**	11.2	10.6 ± 3.5	14.3 ± 8.9	38.8 ± 15.6	81.2
**Lungs**	13.2	9.6 ± 2.5	12.4 ± 3.7	24.8 ± 4.6	49.5
**Muscle**	7.4	9.2 ± 0.9	9.6 ± 1.1	10.0 ± 0.4	10.8
**Ovaries**	16.3	53.0 ± 9.5	42.3 ± 5.7	14.1 ± 0.1	14.2
**Pancreas**	16.3	10.2 ± 1.3	14.3 ± 3.6	20.6 ± 0.7	17.7
**Red marrow**	12.3	12.1 ± 0.6	12.0 ± 0.6	10.1 ± 0.3	18.4
**Osteogenic Cells**	21.9	8.4 ± 3.2	10.5 ± 3.7	12.9 ± 3.3	20.5
**Skin**	9.9	4.4 ± 1.5	5.4 ± 1.8	8.3 ± 0.6	7.8
**Spleen**	15.3	6.9 ± 2.4	8.6 ± 2.0	12.9 ± 3.3	12.6
**Testes**	12.8	11.8 ± 0.3	10.2 ± 2.0	7.9 ± 0.1	8.4
**Thymus**	13.7	3.4 ± 2.7	5.4 ± 2.7	8.3 ± 0.6	11.8
**Thyroid**	12.8	3.0 ± 2.8	5.0 ± 3.1	7.8 ± 0.7	9.4
**Urinary bladder**	15.6	185.6 ± 78.0	74.1 ± 41.9	61.3 ± 3.3	42.0
**Uterus**	16.9	31.7 ± 4.7	23.5 ± 1.4	14.9 ± 0.1	14.7
**Total body**	12.4	11.0 ± 0.6	11.3 ± 0.6	11.5 ± 0.0	-
	**μSv/MBq**				
**Effective dose equivalent (EDE)**	15.2	105.3 ± 32.9	98.0 ± 46.8	69.5 ± 1.4	-
**Effective dose (ED)**	13.8	123.0 ± 27.4	93.7 ± 19.0	18.9 ± 1.4	22.5

To estimate the human dosimetry from the Formosan rock monkey whole-body PET images data, the organ weight and body mass were used for allometric scaling after obtaining the residence time of each organ for each subject. However, the organ weights of Formosan rock monkey are unavailable; since rhesus monkeys (*Macaca mulatta*) are genetically similar to Formosan rock monkeys (*Macaca cyclopis*) [65, 66], we used organ weights of rhesus monkeys to calculate human dosimetry. The results (Table 4) showed that the human estimated dosimetry of [^18^F]T807 in most organs ranged between 6 and 764 μGy/MBq. The critical organ of [^18^F]T807 was the gallbladder. The estimated effective dose of [^18^F]T807 extrapolated from monkeys was 19 μSv/MBq (n = 2), which was in agreement with human data from Choi’s study [64] and was comparable to those of many ^18^F-labeled PET tracers [67, 68]. The guidelines on radiation exposure for human subjects involved in research studies varied internationally. Based on the guidelines of National Institutes of Health (NIH), the maximum exposure is 50 mSv of effective dose per year for a research subject [69]. On the other hand, under the guidelines of the European commission, the intermediate risk levels in adults will be equivalent to an effective dose range of 1–10 mSv per annum [69].

The toxicity of T807 has not been reported. However, the amounts of T807 that have been injected into the subjects in previous [^18^F]T807 human studies have been reported to be 0.2~0.6 μg (Table 5) [12, 34, 64, 70–75]. Thus, the amount of [^18^F]T807 (185 ± 18.5 MBq or ≤ 0.32 μg of T807) that will be injected to the subjects of our clinical study is reasonable and within the safety range.

**Table 5:** Safety summary of the [^18^F]T807 injection.

Reference	Subject type	Subject number	Activity injected (MBq)	Specific Activity (GBq/μmol, EOS)	Amount of non-radioactive T-807 injected (μg)	Safety Note
[12]	3HC, 1 MCI, 2 AD	6	370	37	< 2.63	Yes
[70]	1 HC	1	190	222 ± 52	0.22	Yes
[34]	56 HC, 13 MCI, 6AD 19 MCI/AD	75	333~407	216 ± 60	< 0.50	-
[64]	6 HC	6	268 ± 31	342 ± 192	0.21	Yes
[71]	10 HC, 6 PSP, 6PD	22	175~183	240–560	< 0.20	-
[72]	8 HC, 8 AD	16	303~341	> 200	< 0.44	-
[73]	5 HC, 5 AD	10	225 ± 16	93~272	< 0.63	-
[74]	21 HC, 3 MCI	24	303~341	216± 60	< 0.41	-
[75]	59 HC, 61 AD/MCI	120	280 ± 37	> 250	< 0.29	-
**Total**		280	175~407	37~560	0.20~0.63	-
This Study	AD	1	185	151	0.32 μg	Yes

### PET/CT imaging of the [^18^F]T807 injection in an AD patient

**Fig 9:**
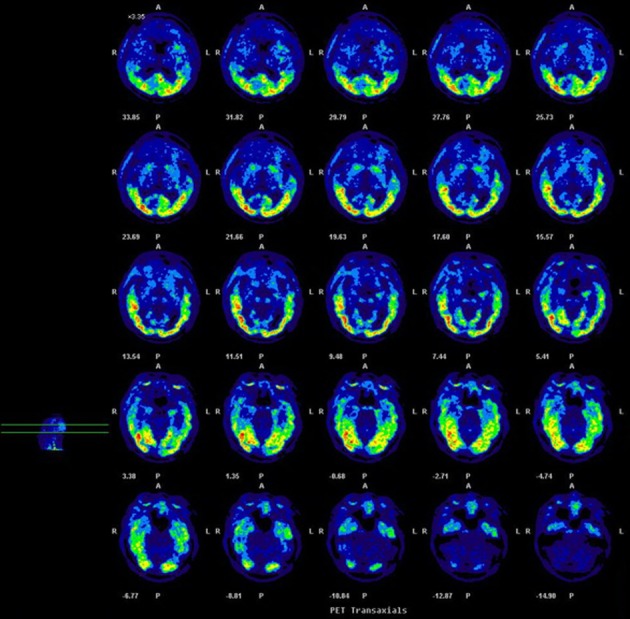
Representative axial PET images of [^18^F]T807 of an AD patient (male, age = 61, MMSE = 24).

Since the GMP-compliant [^18^F]T807 injection synthesized by this one-pot two-step method seems to be safe for human study, we carried out the PET/CT Imaging of the [^18^F]T807 injection in an AD patient (Fig 9). The results showed that [^18^F]T807 did not produce overt adverse effects clinically, and had considerable uptake in the lateral temporal lobe, mesial temporal lobe, hippocampal area, and occipital lobe, which is consistent with the results reported in the literature [33].

## Conclusions

With this one-pot two-step synthesis of the [^18^F]T807 injection using a FX_FN_ module, a GMP-compliant [^18^F]T807 injection can be automatically produced with high reproducibility and high quality. PET imaging and radiation dosimetry evaluation in mice and Formosan rock monkeys suggested that the [^18^F]T807 injection synthesized by this method is suitable for use in human PET imaging studies. Preliminary study in an AD patient showed that [^18^F]T807 bound to tau protein, and clinical studies are in progress for imaging tauopathies in humans.

## Supporting information

S1 Fig The identity of t-Boc protected precursor (3) of [^18^F]T807 was confirmed by ^1^H-NMR. ^1^H-NMR (400 MHz, CDCl_3_): δ 1.80 (s, 9H),7.71 (dd, *J* = 8.0, 1.6 Hz, 1H), 8.15 (d, *J* = 5.8 Hz, 1H), 7.23 (d, *J* = 8.1 Hz, 1H), 8.33 (dd,*J* = 8.5, 2.3 Hz,1H), 8.40 (d, *J* = 8.0 Hz, 1H), 8.70 (d, *J* = 5.8Hz, 2H), 8.97 (d, *J* = 2.0Hz, 1H), 9.3 (d, *J* = 0.6Hz, 1H).
(TIF)

S2 Fig The identity of t-Boc protected precursor (3) of [^18^F]T807 was confirmed by ^13^C-NMR. ^13^C-NMR (100MHz, CDCl_3_): δ 28.5, 86.0, 111.3, 115.87, 118.0, 121.2, 121.3, 123.4, 124.9, 135.2, 138.2, 139.3, 142.8, 143.2, 144.1, 147.5, 148.7, 150.4, 155.9.
(TIF)

S3 Fig The identity of t-Boc protected precursor (3) of [^18^F]T807 was confirmed by mass spectra. ESIHRMS: Calcd for C_21_H_18_N_4_O_4_Na [M+Na]^+^, 413.1220; found, 413.1221.
(TIF)

S4 Fig The chemical purity of 3 was determined by RP-HPLC. RP-HPLC (Phenomenex Gemini C_18_, 5μm, 4.6×250 mm, MeCN/0.05M NH4OAc (7/3), 254nm, 1mL/min, 10μl)
(TIF)
