# Supplementary figures and images for: An one-pot two-step automated synthesis of [18F]T807 injection, its biodistribution in mice and monkeys, and a preliminary study in humans
Source: PLoS One. 2019 Jul 1;14(7):e0217384. doi: 10.1371/journal.pone.0217384 (PMC6602418; doi:10.1371/journal.pone.0217384)

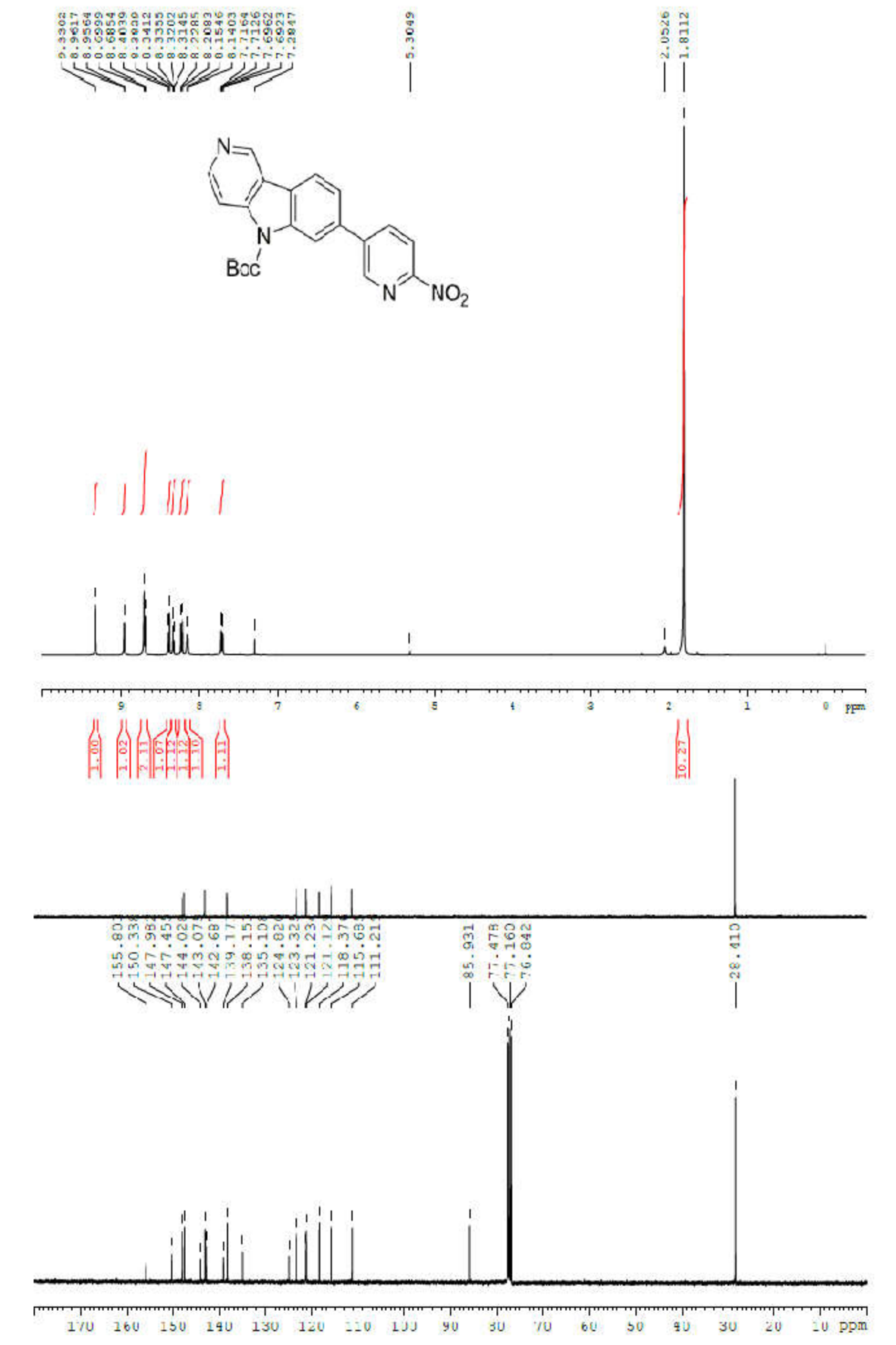

Supplement: S1 Fig — 1H-NMR (400 MHz, CDCl3): δ 1.80 (s, 9H),7.71 (dd, J = 8.0, 1.6 Hz, 1H), 8.15 (d, J = 5.8 Hz, 1H), 7.23 (d, J = 8.1 Hz, 1H), 8.33 (dd,J = 8.5, 2.3 Hz,1H), 8.40 (d, J = 8.0 Hz, 1H), 8.70 (d, J = 5.8Hz, 2H), 8.97 (d, J = 2.0Hz, 1H), 9.3 (d, J = 0.6Hz, 1H). (TIF) [file pone.0217384.s001.tif]

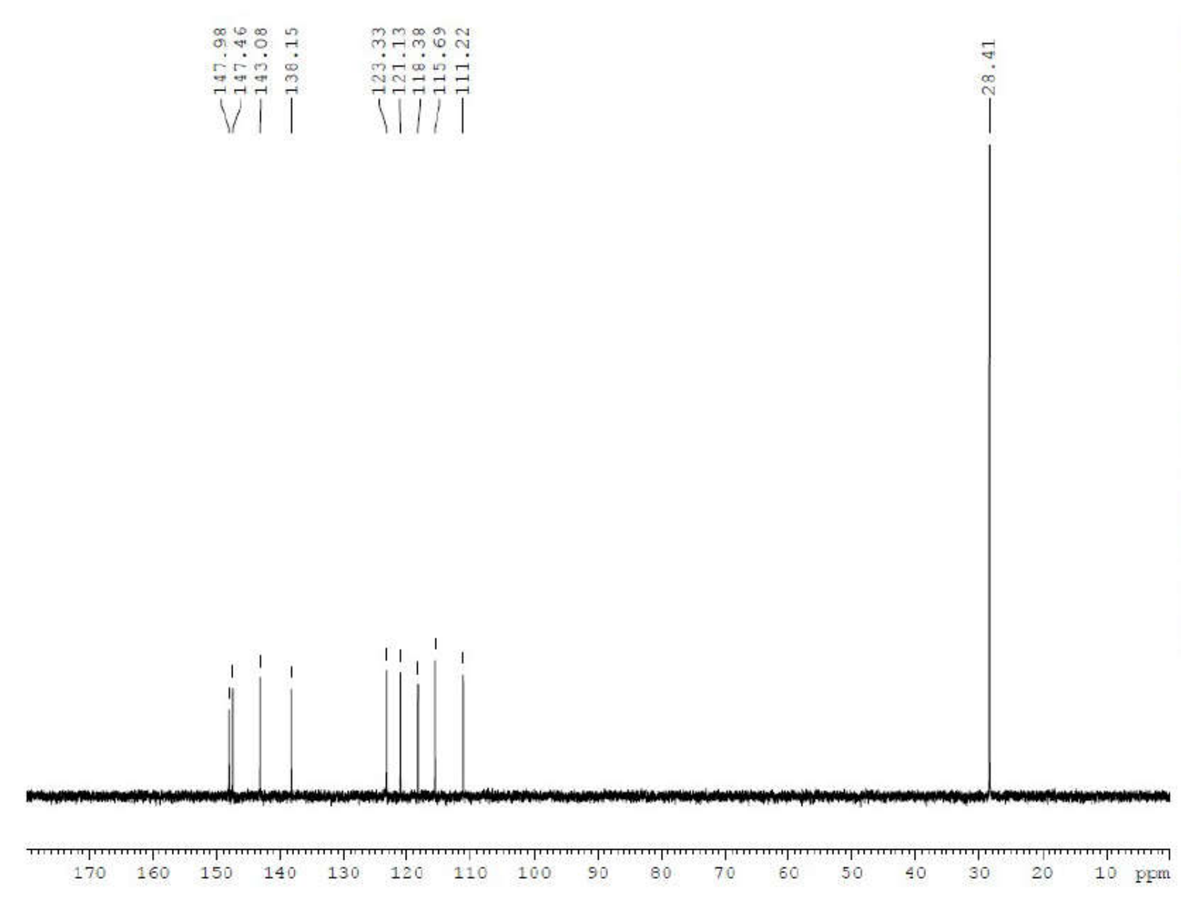

Supplement: S2 Fig — 13C-NMR (100MHz, CDCl3): δ 28.5, 86.0, 111.3, 115.87, 118.0, 121.2, 121.3, 123.4, 124.9, 135.2, 138.2, 139.3, 142.8, 143.2, 144.1, 147.5, 148.7, 150.4, 155.9. (TIF) [file pone.0217384.s002.tif]

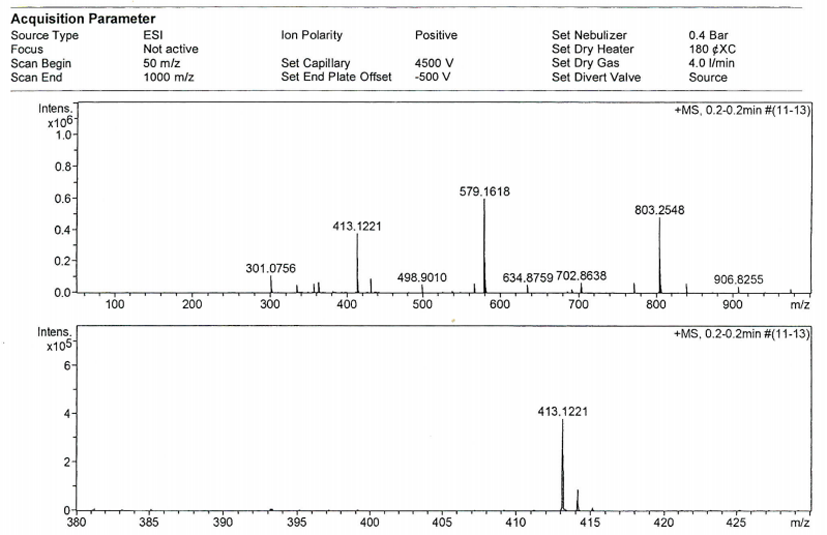

Supplement: S3 Fig — ESIHRMS: Calcd for C21H18N4O4Na [M+Na]+, 413.1220; found, 413.1221. (TIF) [file pone.0217384.s003.tif]

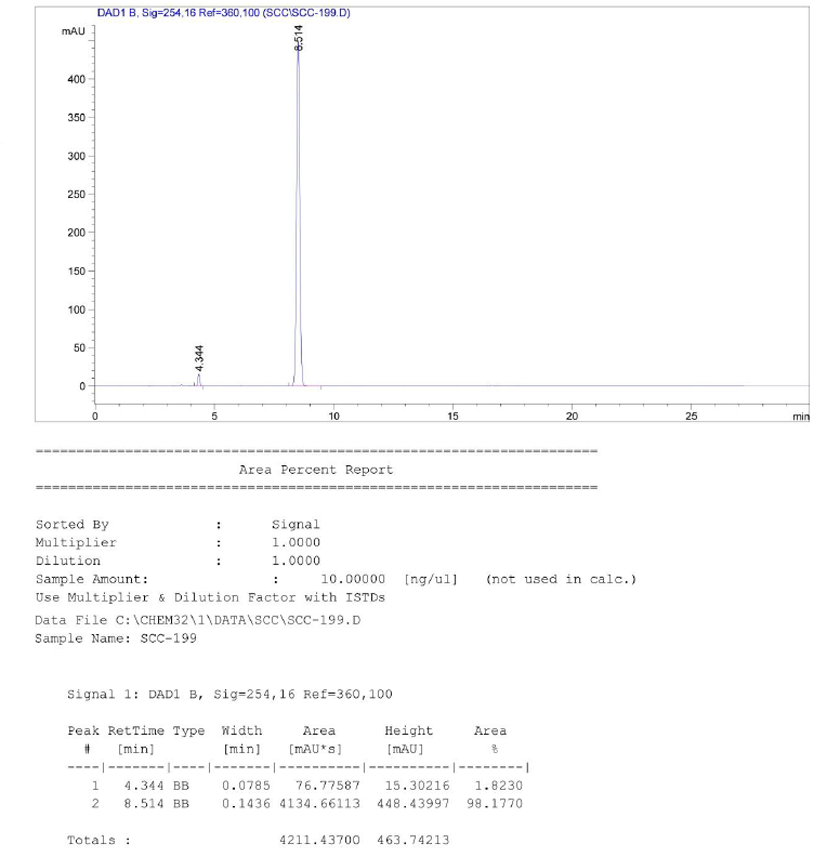

Supplement: S4 Fig — RP-HPLC (Phenomenex Gemini C18, 5μm, 4.6×250 mm, MeCN/0.05M NH4OAc (7/3), 254nm, 1mL/min, 10μl) (TIF) [file pone.0217384.s004.tif]
